# Long-Term Outcomes After Arterial Switch Operation for dextro-Transposition of the Great Arteries—30-Year Single-Center Experience

**DOI:** 10.3390/jcm14093160

**Published:** 2025-05-02

**Authors:** Johanna Schlein, Clemens Ungerböck, Daniela Tertschnig, Alexandra Kaider, Barbara Karner, Clemens Atteneder, Erhan Urganci, Paul Werner, Eva Base, Peter Murin, Daniel Zimpfer

**Affiliations:** 1 Department of Cardiac and Thoracic Aortic Surgery, Medical University of Vienna, 1090 Vienna, Austria; 2 Department of Pediatrics and Adolescent Medicine, Division of Pediatric Pulmonology, Allergology and Endocrinology, Medical University of Vienna, 1090 Vienna, Austria; 3 Center for Medical Data Science, Institute of Clinical Biometrics, Medical University of Vienna, 1090 Vienna, Austria; 4 Department of Anaesthesia, Intensive Care Medicine and Pain Medicine, Division of Cardiac Thoracic Vascular Anaesthesia and Intensive Care Medicine, Medical University of Vienna, 1090 Vienna, Austria

**Keywords:** arterial switch operation, dextro-transposition of the great arteries, correction of dextro-transposition of the great arteries

## Abstract

**Background/Objectives**: The arterial switch operation (ASO) has been performed in the neonatal period as an anatomical correction for d-transposition of the great arteries since the 1980s. As the population of adult congenital heart disease patients grows, it is essential for healthcare providers across various disciplines to comprehend the complexities of these patients. We report on outcomes up to the third decade after ASO. **Methods**: All patients who underwent ASO from May 1985 to December 2020 were included, and a retrospective chart review with follow-up until December 2021 was conducted. Additionally, vital status verification (90.3% complete) was performed through the national health insurance. Survival analysis and competing risk analysis were performed to determine outcomes in the third decade after ASO. **Results**: One-hundred-ninety-five patients (72.3% male; median age at surgery 6 days; median weight at surgery 3.4 kg) underwent ASO from May 1985 to December 2020. Patients with a prenatal diagnosis showed a lower rate of preoperative cardiac decompensation (*p* = 0.017). Early in-hospital mortality was 8.7%, and no early in-hospital deaths occurred in the study era from 2006 to 2020. Four late deaths occurred, and the Kaplan–Meier estimated survival of the 178 hospital survivors was 98.3% ± 1.2% at 10 years, 96.8% ± 1.9% at 20 years and 92.4% ± 4.7 at 30 years. The cumulative incidence of left ventricular outflow tract (LVOT) reoperation after ASO was 1.3% at 10 years, 3.4% at 20 years and 11.3% at 30 years. The cumulative incidence of right ventricular outflow tract (RVOT) reoperation after ASO was 7.2% at 10 years and 17.5% at 20 and 30 years. **Conclusions**: Overall long-term survival of the hospital survivors is good. The reoperation rate on the LVOT is favorable. Percutaneous interventions, if feasible after the Lecompte maneuver, might pose an option to delay the more common reoperations on the RVOT, though further studies are needed to determine their long-term impact.

## 1. Introduction

D-Transposition of the great arteries (d-TGA) is a cyanotic complex congenital heart defect and has an incidence of 0.3 per 1000 livebirths [1]. In d-TGA, the aorta arises from the morphological right ventricle, while the pulmonary artery originates from the morphological left ventricle, resulting in ventriculo-arterial discordance. The circulation in d-TGA is ductus-dependent and differs regarding other shunts at septal level such as persistent foramen ovale, atrial septal defect and/or ventricular septal defect. These patients require urgent intervention shortly after birth. Prostaglandins are used to keep the ductus arteriosus open. Depending on the severity of cyanosis, a persistent foramen ovale or restrictive atrial septal defect may be dilated using a Rashkind procedure, in which the septal defect is enlarged using a balloon. The first successful anatomical correction of d-TGA via the arterial switch operation (ASO) was reported by Adib Jatene in 1975 [2,3] and, throughout the 1980s and 1990s, replaced atrial baffle operations (the Senning operation since 1957 [4] or the Mustard operation since 1963 [5]), as earlier in the 1970s concerns regarding the long-term function of the systemic right ventricle as well as the incidence of arrythmias following atrial baffle operations arose [3]. Nowadays, the early ASO patients are reaching their third and fourth decades of life. We analyzed outcomes regarding survival and reoperation rates after ASO.

As the number of adult congenital heart disease patients increases, it is crucial for specialists, general practitioners and healthcare providers in various fields to understand the complexities of congenital heart disease. Given the lifelong nature of care, continuous monitoring and appropriate management strategies are essential, particularly as patients transition from pediatric to adult care. Continuous monitoring of this patient cohort at a specialized center is warranted. The possibility of a 30-year follow-up is only facilitated by an interdisciplinary approach at a pediatric heart center and the subsequent transfer to a specialized outpatient clinic for adults with congenital heart disease.

## 2. Patients and Methods

### 2.1. Patients

All patients who underwent ASO from 10 May 1985 to 31 December 2020 were included in this retrospective study, and follow-up data were included until 12 December 2021. Additionally, vital status verification (90.3% complete) was performed through national health insurance. Nineteen patients (9.7%; 19/195) could not be verified as they had been referred for ASO from foreign centers and were censored at their last documentation at the study center. Median follow-up time was 13.9 years (IQR 4.8–25.2 years), with the longest follow-up time of 34.6 years. The local ethics committee gave a positive vote for this retrospective study (Ethics Committee submission number: 1062/2021; approval granted on 3 March 2021). Due to the retrospective nature of the study and the use of anonymized clinical data, the requirement for individual patient consent was formally waived by the committee.

### 2.2. Management

#### 2.2.1. Antenatal Management

A referral for fetal echocardiography at the specialized center might be decided upon the generally available organ screening in the second trimester or based on family history for congenital heart disease. A pregnancy with a fetus with d-TGA today is planned either for cesarean section or vaginal delivery, depending on the presence of a shunt (atrial septum and/or ventricular septum) and the shunt volume, which is quantified antenatally to plan the mode of delivery and to determine the need for an immediate Rashkind procedure and plan for by-standing procedural equipment and personnel already during delivery.

#### 2.2.2. Surgical Technique

In the ASO, an anatomical correction is performed by switching the great arteries to their anatomical correct positions. The ASO is conducted during the neonatal period, as delaying the procedure increases the risk of the left ventricle being unable to generate sufficient systemic pressure following the decrease in pulmonary vascular resistance. Additionally, the right ventricle can remain functionally a sub-pulmonary ventricle, avoiding the systemic pressure load.

The surgery is performed via sternotomy and on cardiopulmonary bypass. A pericardial patch, which is fixed in glutaraldehyde or left native, is procured. Initially, the ascending aorta and pulmonary arteries are fully mobilized. Arterial cannulation is performed high and anterior to the truncal descent, and venous cannulation is performed bicavally. Cardiopulmonary bypass is established, and the ductus arteriosus is ligated and subsequently divided. After aortic clamping and administration of cardioplegia, the aorta is transected distal to the coronary ostia. Prior to excision, the coronary arteries are carefully evaluated from the coronary ostia to assess for any intramural course to avoid accidental injury. After isolating the coronary ostia as coronary buttons, the coronary branches are mobilized. The pulmonary artery is transected proximally to the bifurcation. The reimplantation of the coronary buttons into the neo-aortic root is a critical step, requiring meticulous technique to ensure that the coronary vessels are sutured without tension or kinking. Improper suturing can result in vessel distortion, which may lead to stenosis and subsequent myocardial ischemia, necessitating revision. An end-to-end anastomosis of the neo-aorta is then performed. The distal pulmonary artery is repositioned anteriorly to the aorta using the Lecompte maneuver and secured by reclamping the aortic clamp. Following this maneuver, the pulmonary bifurcation is positioned anterior to the aorta. During rewarming and under released coronary perfusion, the continuity of the neopulmonary root is restored using the pericardial patch, which is then anastomosed end-to-end with the distal neopulmonary artery.

In the setting of the side-to-side vessel position of Taussig-Bing anomaly, meticulous dissection of the arteries is needed for sufficient mobilization, and in most cases the Lecompte maneuver is not performed.

#### 2.2.3. Follow-Up Management

Patients have routine appointments including an echocardiography at the pediatric cardiology outpatient clinic or at the outpatient clinic for adults with congenital heart disease at least annually. In the setting of neo-aortic root dilatation, an angio-computer tomography is performed half-annually, and surgery is considered in patients with a neo-aortic root diameter > 55 mm (aortic height and aortic size index are measured additionally) or aortic diameter growth rate > 3 mm/year in patients with a neo-aortic root diameter > 50 mm. Cardiac magnetic resonance imaging is performed for quantitative assessment of valve regurgitation to determine whether a valve-sparing root replacement is feasible or whether neo-aortic valve replacement using a composite valve graft (Bentall procedure) is required in the setting of significant regurgitation. Stenting or surgery of pulmonary branch stenosis is considered regardless of symptoms in the setting of reduced lung perfusion (flow distribution between the left and right lungs) and/or diameter narrowing over 50% assessed by cardiac magnetic resonance imaging (also to rule out compression in the setting of a dilatated neo-aortic root), as echocardiographic assessment is limited due to the retrosternal anterior position of the pulmonary bifurcation and the branches after the Lecompte maneuver. The occurrence of new ventricular dysfunction (global or regional) and/or arrhythmias entail full coronary work-up (coronary computer tomography and stress magnetic resonance imaging to assess myocardial perfusion in adults and in selected cases in adolescents). Regardless of age and time since ASO at the suspicion of acute myocardial ischemia, coronary angiography is warranted to evaluate ostial/proximal stenoses of the reimplanted coronary arteries. Screening in terms of coronary computer tomography should be considered in this cohort as patients become older and coronary artery disease risk becomes more present.

### 2.3. Definitions

Early in-hospital mortality was defined as death occurring as in-hospital death after surgery during index hospitalization. Death was defined as all-cause mortality. Coronary anatomy was classified according to the modified Leiden Convention [6]. Coronary anatomy other than Leiden Convention type A (1LCx–2R) was defined as a coronary anomaly. Reoperation rates were evaluated for the left ventricular outflow tract (LVOT) and the right ventricular outflow tract (RVOT) solitarily, as well as for any ASO-related reoperation including outflow tract reoperations and reoperations related to the reinserted coronary arteries combined. Percutaneous reinterventions (pulmonary artery dilatations with/without stenting; pulmonary valve balloon valvuloplasty; percutaneous pulmonary valve replacement) are described separately. The study endpoints were compared between surgical eras: 1985 to 1995 (28.2%; 55/195), 1996 to 2005 (24.6%; 48/195) and 2006 to 2020 (47.2%; 92/195).

### 2.4. Statistical Analysis

The Chi-square test or Fisher’s exact test, in the case of expected cell frequencies less than 5, were used for comparisons of categorical variables. Continuous variables were compared between groups using the Mann–Whitney U test (2 groups) and the Kruskal–Wallis test (3 groups). The median follow-up time was calculated by the inverse Kaplan–Meier method [7]. Kaplan–Meier calculation was used to report on survival probabilities, and univariable Cox proportional hazards regression models were performed to investigate the potential association of risk factors (surgical era, coronary anomaly and d-TGA with intact ventricular septum) with survival. Potential associated anatomical and procedural variables (surgical era, d-TGA with intact ventricular septum, coronary anomaly, concomitant surgery for aortic arch obstruction) with early in-hospital mortality were analyzed with the Chi-square test or Fisher’s exact test. Competing risk analysis was performed to evaluate the time-related occurrence of ASO-related reoperation by the estimation of the cumulative incidence function, where death was considered a competing event. Gray’s test was performed to test for differences between cumulative incidence functions. Univariable Fine and Gray proportional subdistribution hazards models [8] were performed to evaluate potential associations of independent anatomical and procedural variables with reoperation (surgical era, d-TGA with intact ventricular septum, coronary anomaly, concomitant surgery for aortic arch obstruction, respectively). Statistical significance was set at *p* < 0.05. Data were analyzed using the software package SPSS^®^ 29 (IBM Corp., Chicago, IL, USA) and SAS 9.4 (SAS Institute Inc., Cary, NC, USA).

## 3. Results

### 3.1. Patient and Perioperative Characteristics

From May 1985 to December 2020, 195 patients (72.3%; 141/195 male) underwent ASO. Patient and perioperative characteristics with regard to the surgical eras are seen in Table 1. Patients born prematurely (8.2%; 16/195) had a significantly higher (*p* = 0.009) rate of preoperative invasive ventilation than patients born at full term. The preoperative clinical status of children with prenatal (13.8%; 27/195) and postnatal diagnosis (86.1%; 168/195) showed a significant difference in the rate of preoperative cardiac decompensation (*p* = 0.017), early administration of prostaglandin (*p* = 0.005) and age at ASO (*p* = 0.002) (Supplemental Table S1).A Rashkind procedure was performed in 51.3% of all patients (51.3%; 100/195), with a significantly higher rate observed between 1996 and 2005, when 75% of patients (75%; 36/48) underwent the procedure. There was a significant difference in the rate of concomitant surgical correction of aortic arch pathologies during the ASO (*p* = 0.014) between surgical eras with 16.3% patients (15/92) receiving concomitant aortic arch repair in the era of 2006 to 2020.

### 3.2. Early Outcome

Early postoperative outcomes with respect to the surgical eras are seen in Table 2. Overall, early in-hospital mortality was 8.7% (17/195), and no early in-hospital deaths occurred in the era from 2006 to 2020. Early in-hospital mortality differed significantly between the surgical eras (*p* < 0.001) but was not associated with intact ventricular septum (*p* = 0.173) or concomitant aortic arch surgery during ASO (*p* = 0.688) or coronary anomaly (*p* = 0.567). Causes of deaths are given in Supplemental Table S2. Six patients (3.1%; 6/195) could initially not be weaned from ventilation due to aortopulmonary collaterals and underwent coiling of the collaterals in the early postoperative setting.

### 3.3. Follow-Up

#### 3.3.1. Survival

**Figure 1:**
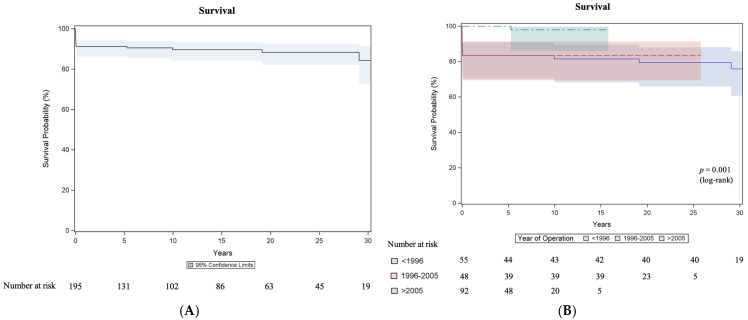
Survival after arterial switch operation. (**A**) Kaplan–Meier estimated survival curve with 95% confidence interval (CI). (**B**) Kaplan–Meier estimated survival curves with 95% CI comparing surgical eras: 1985–1995, 1996–2005 and 2006–2020.

A total of 21 deaths (17 early in-hospital deaths and 4 late deaths) occurred, and Kaplan–Meier estimated survival was 89.6% (95% CI 84.1–93.3) at 10 years, 88.3% (95% CI 82.1–92.5) at 20 years and 84.3% (95% CI 72.5–91.3) at 30 years (Figure 1A). As seen in Figure 1B, Kaplan–Meier estimated survival differed significantly (*p* = 0.001) between the surgical eras: 1985–1995 (81.5% (95% CI 68.3–89.6) at 10 years, 79.5% (95% CI 66.0–88.1) at 20 years, 75.9% (95% CI 60.7–85.9) at 30 years), 1996–2005 (83.3% (95% CI 69.4–91.3) at 10, 20 and 30 years) and 2006–2020 (97.9% (95% CI 86.1–99.7) at 10 and 15 years). The causes of late deaths are seen in Supplemental Table S2. In the univariable Cox proportional hazards regression analysis, the surgical era was associated with mortality (HR (1996–2005 vs. 1985–1995) 0.82, 95% CI 0.33–2.04; HR (2006–2020 vs. 1985–1995) 0.06, 95% CI 0.01–0.44; *p* = 0.023)). However, neither the presence of a ventricular septal defect (HR 1.7, 95% CI 0.7–4.1; *p* = 0.212) nor the presence of coronary anomalies (HR 0.7, 95% CI 0.2–2.; *p* = 0.488) showed a significant association with mortality.

Four late deaths (2.2%; 4/178) occurred, and the Kaplan–Meier estimated survival of the 178 hospital survivors was 98.3% ± 1.2% at 10 years, 96.8% ± 1.9% at 20 years and 92.4% ± 4.7 at 30 years.

#### 3.3.2. Left Ventricular Outflow Tract Reoperation

**Figure 2:**
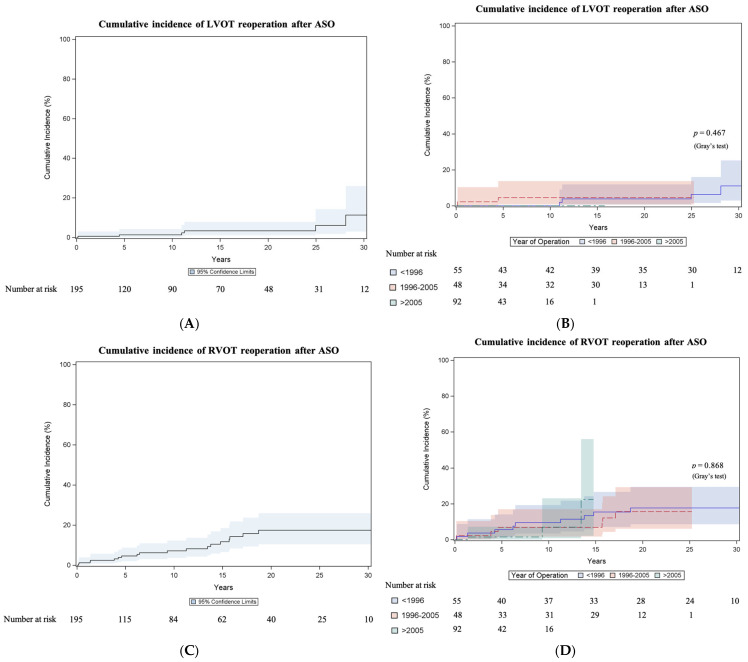
Cumulative incidence of reoperation after arterial switch operation. (**A**) Cumulative incidence curve of left ventricular outflow tract (LVOT) reoperation after arterial switch operation (ASO). Curve with 95% confidence interval (CI). (**B**) Cumulative incidence curves of LVOT reoperation after ASO comparing surgical eras: 1985–1995, 1996–2005 and 2006–2020. Curves with 95% CI. (**C**) Cumulative incidence curve of right ventricular outflow tract (RVOT) reoperation after ASO. Curve with 95% CI. (**D**) Cumulative incidence curves of RVOT reoperation after ASO comparing surgical eras: 1985–1995, 1996–2005 and 2006–2020. Curves with 95% CI.

Cumulative incidence of LVOT reoperation after ASO was 1.3% (95% CI 0.3–4.3) at 10 years, 3.4% (95% CI 1.1–7.9) at 20 years and 11.3% (95% CI 3–26) at 30 years (Figure 2A). The incidence did not differ (Gray test *p* = 0.467) between surgical eras (Figure 2B). Six patients (3.1%; 6/195) underwent at least one reoperation on the LVOT. These included two patch plasties at the aortic anastomosis site performed 11 years and two post-ASO, respectively, one Tirone David procedure performed 25 years post-ASO and three aortic valve replacements (one in a bicuspid valve). One patient required a second LVOT reoperation 13 years after the first reoperation due to relative stenosis of her 19 mm mechanical valve. The valve was replaced to a 21 mm mechanical valve at the age of 17 years.

#### 3.3.3. Right Ventricular Outflow Tract Reoperation

The cumulative incidence of RVOT reoperation after ASO was 7.2% (95% CI 3.6–12.3) at 10 years and 17.5% (95% CI 10.4–26) at 20 and 30 years (Figure 2C). The incidence did not differ (Gray test *p* = 0.868) between surgical eras (Figure 2D). The results of the Fine and Gray regression analyses for anatomical and procedural variables associated with RVOT reoperation are shown in Supplemental Table S3. Eighteen patients (9.2%; 18/195) required at least one reoperation on the RVOT. These included twelve patch plasties on the pulmonary arteries, three pulmonary valve repairs combined with pulmonary patch plasty and one right pulmonary artery conduit. Two pulmonary valve replacements were performed utilizing a 23 mm decellularized homograft after 15 years in one case and a 25 mm bioprosthetic valve sewn into a vascular prosthesis after 9 years in another case. A second RVOT reoperation was required in seven patients and a third RVOT reoperation in two patients.

#### 3.3.4. Right Ventricular Outflow Tract Percutaneous Reinterventions

Seventeen pulmonary artery dilatations or dilatations with additional stenting were performed in 12 patients (6.2%; 12/195). Ten pulmonary valve interventions were performed in 7 patients (3.6%; 7/195): seven balloon valvuloplasties, one balloon dilatation of a homograft and one implantation of a Melody valve.

#### 3.3.5. Any Reoperation Related to Arterial Switch Operation

**Figure 3:**
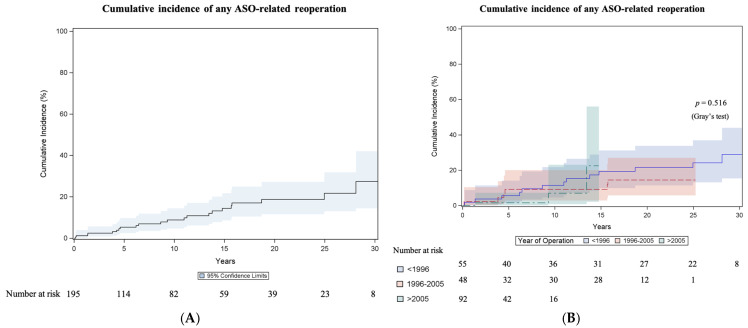
Cumulative incidence of any reoperation related to arterial switch operation. (**A**) Cumulative incidence curve of any arterial switch operation (ASO)-related reoperation. Curve with 95% confidence interval (CI). (**B**) Cumulative incidence curves of any ASO-related reoperation comparing surgical eras: 1985–1995, 1996–2005 and 2006–2020. Curves with 95% CI.

Twenty-two patients (11.3%; 22/195) had at least one ASO-related reoperation (outflow tract reoperations and reoperations related to the coronary arteries), and the cumulative incidence of any ASO-related reoperation was 8.8% (95% CI 4.8–14.4) at 10 years, 18.7% (95% CI 11.6–27.2) at 20 years and 27.4% (95% CI 14.5–42.1) at 30 years (Figure 3A). The incidence did not differ (Gray test *p* = 0.516) between surgical eras (Figure 3B). The results of the Fine and Gray regression analyses for anatomical and procedural variables associated with any ASO-related reoperation are seen in Supplemental Table S4. Two late coronary artery bypass graft surgeries (left internal thoracic artery to left anterior descending artery) were performed after 9 and 15 years, respectively. Coronary ostial plasty was performed in a 14-year-old patient due to acute myocardial ischemia resulting from ostial obstruction.

#### 3.3.6. Arrhythmias and Endocarditis

Seven patients (3.6%; 7/195) had documented follow-up visits due to late arrythmias. Three patients required late pacemaker implantation due to sick-sinus-syndrome two months, 13 years and 11 years after ASO, respectively. One patient needed late implantable cardioverter defibrillator implantation 25 years post-ASO after cardiopulmonary resuscitation due to ventricular fibrillation. One patient experienced an endocarditis, which needed pulmonary valve replacement 19 years after ASO.

## 4. Discussion

We found an improvement over time regarding early mortality, need for extracorporeal membrane oxygenation, ventilation time, time of intensive care unit stay and hospital stay. Initially, early mortality was high, and it decreased over time with the evolvement of the perioperative management. Late mortality was low, and reoperation on the RVOT was more common than on the LVOT (Graphical Abstract). Additionally, Supplemental Table S5 summarizes data from the discussed recent single-center outcome studies [9,10,11,12,13] alongside pooled estimates from a benchmark meta-analysis [14]. Reported outcomes in Supplemental Table S5 include overall survival and rates of reoperation.

Early in-hospital mortality (8.7%; 17/195) was comparable to other recent retrospective chart review studies, which included patients starting in the 1980s with rates ranging from 3.3% to 8.8% [10,11,13]. We have seen an era effect regarding early in-hospital mortality (*p* < 0.001) with early in-hospital mortality rates of 16.4% (9/55) from 1985 to 1995, 16.7% (8/48) from 1996 to 2005 and 0% (0/92) from 2006 to 2020. Also, Van der Palen et al. [13] saw a decrease in hospital mortality with an overall hospital mortality of 8.8% (43 patients), with the highest from 1977 to 1987, which decreased to 3.3% between 2000 and 2020. The causes of early in-hospital death (Supplemental Table S2) were similar to those of the mentioned studies [10,11] with Santens et al. [11], who identified cardiogenic shock in the course of myocardial ischemia, sepsis-induced multiple organ failure and pulmonary hypertension as causes of early death.

Regarding long-term survival in the meta-analysis by Morfaw et al. [14], a subgroup analysis suggests a slightly higher survival following ASO in the second surgical era (1998 to 2018) than in the first surgical era (1975 to 1997) in the pooled short-term survival (0–1 year) and pooled medium-term survival (1–20 years), with 93.0% vs. 90.0% and 93.0% vs. 88.0%, respectively, but not in the pooled long-term survival (>20 years), with 81.0% vs. 89.0%. The overall pooled long-term survival rate with 87% (95% CI 80–92) is comparable with our 30-year survival rate of 84.3% and the 30-year survival rate of 89.6% of the Leuven group [11]. In accordance with the era effect of early in-hospital mortality, we have seen differences (*p* = 0.001) between the eras regarding survival: 1985–1995 (81.5% ± 5.3% at 10 years, 79.5% ± 5.5% at 20 years, 75.9% ± 6.3% at 30 years), 1996–2005 (83.3% at 10, 20 and 30 years) and 2006–2020 (97.9% ± 2.1% at 10 and 15 years). We report four late deaths (2.2%; 4/178), and the Kaplan–Meier estimated survival of the 178 hospital survivors was 98.3% ± 1.2% at 10 years, 96.8% ± 1.9% at 20 years and 92.4% ± 4.7 at 30 years, which is similar to the 30-year survival rate of the hospital survivors from the Leiden group [13], with 94.5%.

Survival after ASO improved as surgical, perioperative treatment and neonatal intensive care evolved over the decades. Additionally, the possibility of prenatal diagnosis poses an advancement in the care of patients with d-TGA. Vida et al. [12] report a fetal diagnosis in 33% in their operative cohort with a significant increase in the later study period. Nagata et al. [15] from The Hospital for Sick Children Toronto report that the prenatal diagnosis rates from 2009 to 2014 differed significantly among areas in Ontario ranging from 14% to 72%. Van Velzen et al. [16], who performed a cohort study in the north-west region of the Netherlands including patients born with d-TGA from 2002 to 2012 (n = 144), describe a rate of prenatal diagnosis of 26.4%. Fetal echocardiography at the specialized center is not universally available in Austria but is only performed on referral. In the last two years of the study period from 2018 to 2020, 38.1% of the patients in this operative cohort had a prenatal diagnosis. Consistent with other studies, which show that prenatal diagnosis reduces mortality and morbidity in d-TGA patients [15,16,17,18,19], we have seen improved clinical status, as in our cohort the occurrence of preoperative cardiac decompensation was lower (*p* = 0.017) in patients with prenatal diagnosis compared to patients with postnatal diagnosis. Antenatal management for pregnancies with congenital heart disease suspected in the fetus evolved. Also, prenatal diagnosis allows for early start of prostaglandin administration after birth. Additionally, in selected patients, in which corrective surgery is not immanently possible clinically, the placement of a persistent ductus arteriosus stent poses an advanced option to allow for deferral of surgery. Additionally, the options for perfusionists regarding pediatrics and in the setting of ASO neonatal oxygenators and tubing kits, as well as neonatal size-adequate cannulas for installment of the heart-and-lung machine, emerged and broadened over the last two decades at the beginning of the 2000s. In the earliest study era, in some cases, phases of complete circulatory arrest were needed during surgery. The neuroprotection regimens of today’s heart-and-lung machines have improved. In the recent period, aortic arch pathologies were operated in a one-stage-approach concomitantly with the ASO (15/92; 16.3% from 2006 to 2020), whereas today antegrade cerebral perfusion is performed. D-TGA patients with aortic arch obstruction pose a complex subgroup with higher operative mortality [20,21] and a higher reintervention rate, especially for right-sided obstruction [22]. Also, regarding coronary anatomy and Taussig–Bing anomaly, surgical evolvement over the study period allowed for corrective surgery with an ASO for these patients. In the last study era from 2006 to 2020, 27.2% of patients undergoing ASO had a coronary anatomy other than modified Leiden Convention type A (1LCx–2R). Additionally, 8.7% (8/92) of patients undergoing ASO from 2006 to 2020 had intramural coronary anatomy. In the earlier study period, intramural coronary anatomy was not corrected with ASO, and these patients underwent the atrial switch operation.

The overall reintervention burden after the ASO in the long term is not to be neglected, with a cumulative incidence of any ASO-related reoperation (including three reoperations on the coronary arteries) of 27.4% at 30 years. The reoperation rate on the LVOT was lower, with a cumulative incidence of 11.3% at 30 years, while the reoperation rate on the RVOT was higher, with 17.5% at 30 years. We have seen no era effect for reoperation. The pooled short-term freedom from reoperation (0–1 years) was 93%, the pooled medium-term freedom from reoperation (1–20 years) was 81% and the pooled long-term (>20 years) freedom from reoperation was 78% in the meta-analysis [14], which included studies with the first patients operated on in 1976. The most common sequelae after the ASO are RVOT obstructions, neo-aortic insufficiency and aortic root dilatation [9,10,23,24,25]. RVOT obstruction after ASO primarily occurs at the supravalvular level at the pulmonary trunk/bifurcation and the pulmonary branch arteries [9,10,23,24,25]. Most obstructions are located at the neo-pulmonary anastomotic site and are clearly related to inadequate growth [26], especially in the setting after the Lecompte maneuver, which might cause torsion of the pulmonary arteries. Cleuziou et al. [24] report the development of RVOT obstruction in 11% of patients at a median time of 3.8 years after ASO with a freedom from RVOT reintervention of 83% at 25 years. Percutaneous interventions, such as pulmonary artery dilatation and/or stenting, or pulmonary valve intervention, if technically feasible after the Lecompte maneuver, may represent a management option for RVOT obstruction in selected patients. Though they might delay surgical reoperation, further investigation is warranted to determine their long-term value. Aortic arch anomalies are associated with the risk of development of RVOT, as patients who undergo aortic arch repair with patch material likely have a larger ascending aorta and aortic arch that may lead to additional torsion on the pulmonary bifurcation [24]. Coronary artery obstructions and obstruction of the LVOT are less prevalent [11,23,27]. In concordance with other studies [10,11,13,28], arrhythmias necessitating device implantation or ablative therapy were rare, with 3.6% of patients in our cohort having documented follow-up for arrhythmias.

### 4.1. Study Limitations

In a retrospective study, the related impediments should be considered when discussing long-term outcomes. The comprehensive advancement of ASO in surgical practice and perioperative care may not have been completely accounted for, despite analysis being conducted for different surgical eras. Follow-up time is long (2711 patient-years), and the cohort size is within the larger ones reported regarding ASO outcomes. Additionally, vital status verification was performed and was near-complete (90.3%). Although our study reports on one of the larger ASO cohorts, the sample size remained limited, constraining the statistical power of the analyses. The small number of events limited our ability to perform multivariable analyses and to adjust for possible confounders.

### 4.2. Future Prospects

This study highlights the excellent survival rates of the hospital survivors following the ASO, consistent with findings from other studies. This suggests that the population of adults who underwent the ASO in infancy will continue to grow, eventually surpassing the number of children with the same diagnosis. A better understanding of long-term outcomes post-ASO might lead to surgical modifications during the initial repair to minimize the risk of late complications and to innovations aimed at improving medical management, especially regarding percutaneous treatment options for these patients. As these patients are reaching their forties, and the first ASO patients, their fifties, there will undoubtedly be an increase in the number of patients requiring care and follow-up within the expanding specialized adult congenital heart disease cardiology service.

However, it is essential that the awareness of these patients extends beyond specialized centers. As the population of adolescents and adults who underwent ASO in infancy continues to grow, these individuals will increasingly be encountered across various sectors of the healthcare system, including by general practitioners, in emergency departments and in other specialties requiring care. A heightened awareness of this patient population is crucial to ensure appropriate management and facilitate timely transfer to specialized centers for optimal treatment.

## 5. Conclusions

There was a high perioperative mortality at the beginning of the study period as ASO was introduced, but no early in-hospital deaths were seen in the last study era, 2006 to 2020. Late survival of the hospital survivors after ASO is excellent. The population of patients reaching their thirties and forties after ASO is continuously growing, and healthcare providers should be aware of the complexity of these patients as late complications following ASO may emerge. LVOT reoperation rates show good results, with only 3.1% of patients requiring reoperation. Reoperation on the RVOT is more common and is mainly required at the supravalvular level on the pulmonary arteries. Percutaneous interventions, such as pulmonary artery dilatation and/or stenting, or pulmonary valve intervention, if feasible after the Lecompte maneuver, may represent an option to delay surgical reoperation on the RVOT, though further studies are needed to determine their long-term impact.

## Figures and Tables

**Table 1:** Patient and perioperative characteristics.

Variable	1985–1995 (n = 55)	1996–2005 (n = 48)	2006–2020 (n = 92)	*p*-Value
Number	55	48	92	
Male	38 (69.1)	36 (75)	67 (72.8)	0.783
Age at ASO	7 (5–10)	6 (4–9)	6 (3–8)	0.092
Premature birth (<37 week)	3 (5.5)	8 (16.7)	5 (5.4)	0.062
Birthweight <2500 g	2 (3.6)	3 (6.3)	3 (3.3)	0.655
Prenatal diagnosis of d-TGA	0 (0)	4 (8.3)	23 (25)	<0.001
Ventricular septal defect	11 (20)	18 (37.5)	34 (37)	0.067
Coarctation of the aorta	4 (7.3)	7 (14.6)	14 (15.2)	0.371
Hypoplastic aortic arch	1 (1.8)	4 (8.3)	8 (8.7)	0.234
Coronary anomaly	16 (29.1)	9 (18.8)	25 (27.2)	0.437
Intramural coronary artery	0 (0)	0 (0)	8 (8.7)	0.011
Weight at ASO (kg)	3.1 (2.6–3.6)	3.2 (3.1–3.8)	3.5 (3.1–3.8)	0.035
Height at ASO (cm)	50 (48.7–51.3)	51 (50–54)	50 (49–53)	0.080
BSA_Haycock_ at ASO	0.21 (0.18–0.22)	0.22 (0.21–0.24	0.22 (0.21–0.24)	0.127
Any previous cardiac intervention	18 (32.7)	41 (85.4)	50 (54.3)	<0.001
Rashkind procedure	17 (30.9)	36 (75)	47 (51.1)	<0.001
Persistent ductus arteriosus stent	0 (0)	0 (0)	3 (3.3)	0.334
Cardiopulmonary bypass time	154 (144–170)	158 (140–190)	161 (125–193)	0.790
Aortic cross clamp time	89 (77–102)	82 (66–99)	98 (73–118)	0.065
Concomitant aortic arch surgery	1 (1.8)	4 (8.3)	15 (16.3)	0.014

Values are presented as n, n (%), median (interquartile range). Continuous variables were compared using the independent-sample Kruskal–Wallis test and categorical variables with Chi-square or Fisher’s exact test. ASO, arterial switch operation; BSA, body surface area; d-TGA, dextro-transposition of the great arteries.

**Table 2:** Early postoperative outcomes.

Variable	1985–1995 (n = 55)	1996–2005 (n = 48)	2006–2020 (n = 92)	*p*-Value
Pacemaker implantation	2 (3.6)	1 (2.1)	2 (2.2)	0.847
Ventilation time (days) *	13 (8–100)	8.5 (6–23)	3 (2–5)	<0.001
Intensive care unit stay (days) *	19 (9–100)	16 (9–26)	7 (5–10)	<0.001
Hospital stay (days) *	50 (29–100)	30 (22–51)	16 (13–22)	<0.001
Revision for bleeding	1 (1.8)	2 (4.2)	2 (2.2)	0.716
Drainage for pericardial tamponade	1 (1.8)	5 (10.4)	1 (1.1)	0.019
Revision coronary arteries/ischemia	1 (1.8)	0	1 (1.1)	>0.99
Cardiopulmonary resuscitation	5 (9.1)	2 (4.2)	2 (2.2)	0.158
Hemofiltration/dialysis	1 (1.8)	7 (14.6)	10 (10.9)	0.041
Delayed sternal closure	15 (27.3)	22 (45.8)	34 (37)	0.154
Extracorporeal membrane oxygenation	7 (12.7)	9 (18.8)	0 (0)	<0.001
Early in-hospital death	9 (16.4)	8 (16.7)	0 (0)	<0.001

Values are presented as n, n (%), median (interquartile range). Continuous variables were compared using the independent-sample Kruskal–Wallis test and categorical variables with Chi-square or Fisher’s exact test. * times of patients, who died during surgery or intensive care unit/hospital stay, were set to a maximum of 100 days.

## Data Availability

The dataset analyzed in this study contains sensitive health information from patients with a rare disease. To protect patient privacy and avoid potential re-identification, even after pseudonymization, the data are not publicly available. However, they may be made available upon reasonable request from the corresponding authors, subject to approval by the Ethics Committee of the Medical University of Vienna.

## Supplementary Materials

The following supporting information can be downloaded at: https://www.mdpi.com/article/10.3390/jcm14093160/s1, Table S1: Prenatal diagnosis and preoperative clinical status; Table S2: Early and late deaths after arterial switch operation; Table S3: Fine and Gray subdistribution hazards regression analyses for independent variables associated with right ventricular outflow tract reoperation after arterial switch operation; Table S4: Fine and Gray subdistribution hazards regression analyses for independent variables associated with any reoperation related to arterial switch operation (outflow tract and coronary artery related reoperation). Table S5: Arterial switch operation outcomes.

## Abbreviations

The following abbreviations are used in this manuscript:

ASO	Arterial switch operation
CI	Confidence interval
d-TGA	Dextro-transposition of the great arteries
IQR	Interquartile range
LVOT	Left ventricular outflow tract
RVOT	Right ventricular outflow tract
