# Supplementary material for: Long-Term Outcomes After Arterial Switch Operation for dextro-Transposition of the Great Arteries—30-Year Single-Center Experience
Source: J Clin Med. 2025 May 2;14(9):3160. doi: 10.3390/jcm14093160 (PMC12072194; doi:10.3390/jcm14093160)
Supplement: Supplementary file 1 [file jcm-14-03160-s001.zip › ASO_Supplemental_Table5.pdf]

**Supplemental Table 5. Arterial Switch Operation Outcomes**										
**Single-Center Long-Term Outcomes After Arterial Switch Operation**										
Study (year of publication)	Center/ Country	Study period	Patients (n)	Early mortality	Survival	Survival of hospital survivors	Freedom from reintervention/ reoperation	LVOT reoperation	RVOT reoperation	Mean/Median Follow-up (years)
Vida et al. (2019)	University of Padua, Italy	1987-2018	267	5.6% (1.6% since 2005)	94% at 10y and 93% at 20y	-	87% at 10y 78% at 20y (any reintervention)	-	11% of patients had RVOT-related reintervention s/reoperations	10.2 (median)
Fraser et al. (2020)	Texas Children's Hospital, USA	1995-2018	394	1.3%	98.2% at 5y 97.8% at 10y and 15y	-	94% at 1y, 89% at 10y, 87% at 15y (any reintervention)	-	39% of reintervention s performed on RVOT	10 (median)
Santens et al. (2020)	University Hospitals Leuven, Belgium	1981-2018	318	7.5%	92.1% at 5 y 91.7% at 10y 89.6% at 20y and 89.6% at 30y	mortality rate at 5y 0.4%, at 10y 1.0%, at 15y 2.6% and at 20y 3.9%	91.6% at 5y, 79.2% at 20y (any reintervention)	-	13.5% of hospital survivors reintervened on RVOT	11.1 (mean)
van der Palen et al. (2021)	Leiden & Amsterdam UMC, Netherlands	1977-2020	490	8.8% (3.3 since 2000)		94.5% at 30y	81% at 10y, 76% at 20y, 71% at 30 and 35y (reoperation)	15/83 (18.1%) of reoperations	50/83 (60.2%) of reoperations	15.6 (median)
Fricke et al. (2022)	Royal Children's Hospital, Australia	1983-2015	844	3.3%	95% at 10, 20 and 25y	-	87% at 10y and 77% at 25y (any reintervention)<br><br>89% at 10y and 81% at 25y (reoperation)	Freedom from reoperation on the neoaortic root or neoaortic valve: 96% at 10y, 94% at 20y, and 92% at 25y	-	15 (median)

Schlein et al. (2025)	Medical University of Vienna, Austria	1985-2020 (follow-up until 2021)	195	8.7% (0% since 2006)	89.6% at 10y, 88.3% at 20y and 84.3% at 30y	98.3% at 10y 96.8% at 20y 92.4% at 30y	91.2% at 10y, 81.3% at 20y and 72.6% at 30y (any ASO-related reoperation)	98.7% at 10y, 96.6% at 20y and 88.7% at 30y (LVOT reoperation)	92.8% at 10y, 82.5% at 20 and 30y (RVOT reoperation)	13.9 (median)
**Meta-analytic Benchmark with Pooled Estimates for Outcomes after Arterial Switch Operation**										
Morfaw et al. (2020)	Multi-study pooled analysis	1975-2018	up to 30,186	-	87% at > 20y (4 studies)	-	78% at > 20y (6 studies)	-	-	-
LVOT, left ventricular outflow tract; *RVOT*, right ventricular outflow tract; *y*, years.										
