# Supplementary material for: Long-Term Outcomes After Arterial Switch Operation for dextro-Transposition of the Great Arteries—30-Year Single-Center Experience
Source: J Clin Med. 2025 May 2;14(9):3160. doi: 10.3390/jcm14093160 (PMC12072194; doi:10.3390/jcm14093160)
Supplement: Supplementary file 1 [file jcm-14-03160-s001.zip › ASO_Manuscript_SupplementalTable1.pdf]

**Supplemental Table 1. Prenatal Diagnosis and Preoperative Clinical Status**			
**Characteristic**	**Postnatal diagnosis (n = 168)**	**Prenatal diagnosis (n = 27)**	***p* value**
Premature birth	11 (6.5)	5 (18.5)	0.051
Weight at birth (g)	3370 (3060-3700)	3260 (2920-3486)	0.188
Preoperative cardiac decompensation	50 (29.8)	2 (7.4)	0.017
Preoperative extracorporeal membrane oxygenation	4 (2.4)	0 (0)	> .99
Preoperative intubation/ventilation	76 (45.2)	8 (29.6)	0.147
Early prostaglandin administration	134 (79.8)	27 (100)	0.005
Age at ASO (days)	7 (4-9)	4 (2-7)	0.002
Early in-hospital mortality after ASO	17 (10.1)	0 (0)	0.136
Values are presented as n, n (%), median (interquartile range). Continuous variables were compared using the independent-samples Mann-Whitney *U* test and categorical variables with Chi-square or Fisher's exact test. *ASO,* arterial switch operation.			
