# Supplementary material for: Long-Term Outcomes After Arterial Switch Operation for dextro-Transposition of the Great Arteries—30-Year Single-Center Experience
Source: J Clin Med. 2025 May 2;14(9):3160. doi: 10.3390/jcm14093160 (PMC12072194; doi:10.3390/jcm14093160)
Supplement: Supplementary file 1 [file jcm-14-03160-s001.zip › ASO_Manuscript_SupplementalTable2.pdf]

Supplemental Table 2. Early and Late Deaths After Arterial Switch Operation					
**Number (sex)**	**ASO (year)**	**Age at surgery**	**Diagnosis/ previous intervention**	**Death (postoperative days/years)**	**Cause of death**
**Early deaths**					
1 (m)	1992	10 days	dTGA + VSD	2 days	Right heart failure and cerebellar hemorrhage
2 (m)	1992	8 days	dTGA + CoA	1 days	MOF
3 (m)	1993	54 days	dTGA + VSD + CoA + hypoplastic aortic arch<br>CoA correction	0 days	MOF
4 (m)	1993	10 days	dTGA	7 days	Pulmonary embolism and pulmonary hemorrhage, ARDS, cardiac decompensation
5 (m)	1993	14 days	dTGA + VSD + CoA<br>Rashkind procedure, CoA correction	12 days	Myocardial ischemia due to myocardial infarction
6 (m)	1994	4 days	dTGA + CoA	2 days	Myocardial ischemia due to myocardial infarction
7 (m)	1995	7 days	dTGA<br>Rashkind procedure	16 days	MOF due to sepsis
8 (m)	1995	6 days	dTGA	1 days	Myocardial ischemia due to myocardial infarction
9 (m)	1995	9 days	dTGA + VSD	7 days	MOF, cerebral hemorrhage
10 (m)	1997	6 days	dTGA + VSD<br>Rashkind procedure	7 days	Cardiac decompensation, cerebral hemorrhage
11 (m)	1998	8 days	dTGA	21 days	Cardiac decompensation
12 (m)	1999	5 days	dTGA + VSD<br>Rashkind procedure	18 days	Right heart failure, sepsis
13 (m)	1999	2 days	dTGA + CoA + hypoplastic aortic arch<br>Rashkind procedure	6 days	MOF, DIC

14 (m)	1999	4 day	dTGA + ASD + VSD	0 days	Cardiac decompensation
15 (m)	2000	10 days	dTGA + CoA<br>Rashkind procedure	0 days	Reduced coronary perfusion, cardiac failure
16 (m)	2000	14 days	dTGA + ASD + VSD	14 days	MOF due to sepsis
17 (m)	2000	4 days	dTGA<br>Rashkind procedure	6 days	Myocardial ischemia due to myocardial infarction
**Late deaths**					
18 (m)	1990	2 days	dTGA	19 years	Unknown aetiology
19 (f)	1992	11 days	dTGA + VSD + bicuspid aortic valve	29 years	Unknown aetiology
20 (m)	1995	4 days	dTGA<br>Rashkind procedure	10 years	MOF due to sepsis and ARDS as a result of a Non-Hodgkin lymphoma
21 (m)	2007	13 days	dTGA<br>Rashkind procedure	5 years	Unknown aetiology

ARDS, acute respiratory distress syndrome; *ASD,* atrial septal defect; *CoA*, coarctation of the aorta; *DIC,* disseminated intravascular coagulation; *dTGA,* dextro-transposition of the great arteries; *ECMO,* extracorporeal membrane oxygenation; *MOF,* multiorgan failure; *VSD*, ventricular septal defect.
