# Supplementary material for: Long-Term Outcomes After Arterial Switch Operation for dextro-Transposition of the Great Arteries—30-Year Single-Center Experience
Source: J Clin Med. 2025 May 2;14(9):3160. doi: 10.3390/jcm14093160 (PMC12072194; doi:10.3390/jcm14093160)
Supplement: Supplementary file 1 [file jcm-14-03160-s001.zip › ASO_Manuscript_SupplementalTable3.pdf]

Supplemental Table 3. Fine and Gray Subdistribution Hazards Regression Analyses for Independent Variables Associated with Right Ventricular Outflow Tract Reoperation after Arterial Switch Operation

	**Univariable models**		
**Variable**	**Hazard ratio**	**Confidence interval (95%)**	***p*-value**
Surgical era			0.856
1985-1995*			
1996-2005	0.8	0.3-2.3	
2006-2020	0.7	0.1-3.1	
Ventricular septal defect vs. intact ventricular septum	1.4	0.5-3.8	0.462
Concomitant aortic arch surgery	0.8	0.1-6.3	0.857
Coronary anomaly	0.6	0.2-2.0	0.399

*Reference
