# Supplementary material for: Long-Term Outcomes After Arterial Switch Operation for dextro-Transposition of the Great Arteries—30-Year Single-Center Experience
Source: J Clin Med. 2025 May 2;14(9):3160. doi: 10.3390/jcm14093160 (PMC12072194; doi:10.3390/jcm14093160)
Supplement: Supplementary file 1 [file jcm-14-03160-s001.zip › ASO_Manuscript_SupplementalTable4.pdf]

Supplemental Table 4. Fine and Gray Subdistribution Hazards Regression Analyses for Independent Variables Associated with Any Reoperation Related to Arterial Switch Operation (Outflow Tract and Coronary Artery related Reoperation)

	**Univariable models**		
**Variable**	**Hazard ratio**	**Confidence interval (95%)**	***p*-value**
Surgical era			0.465
1985-1995*			
1996-2005	0.6	0.2-1.7	
2006-2020	0.5	0.1-2.0	
Ventricular septal defect vs. intact ventricular septum	1.1	0.4-2.9	0.829
Concomitant aortic arch surgery	0.7	0.1-5.4	0.735
Coronary anomaly	0.9	0.3-2.3	0.817

*Reference
